# Rheumatologische Versorgung älterer Menschen: Jahresbericht 2026 aus der Kerndokumentation der regionalen kooperativen Rheumazentren

**DOI:** 10.1007/s00393-026-01847-9

**Published:** 2026-07-16

**Authors:** Katinka Albrecht, Katja Thiele, Tobias Alexander, Martin Aringer, Jacqueline Detert, Thorsten Eidner, Martin Feuchtenberger, Jörg Henes, Kirsten Karberg, Uta Kiltz, Benjamin Köhler, Udo Schneider, Jutta G. Richter, Guido Schäfer, Susanna Späthling-Mestekemper, Mirko Steinmüller, Silke Zinke, Anja Strangfeld, Johanna Callhoff

**Affiliations:** 1 Programmbereich Epidemiologie und Versorgungsforschung, Deutsches Rheuma-Forschungszentrum (DRFZ) Berlin, Charitéplatz 1, 10117 Berlin, Deutschland; 2 Medizinische Klinik mit Schwerpunkt Rheumatologie und Klinische Immunologie, Charité – Universitätsmedizin Berlin, Berlin, Deutschland; 3 Deutsches Rheuma-Forschungszentrum (DRFZ) Berlin, Berlin, Deutschland; 4 Bereich Rheumatologie, Medizinische Klinik und Poliklinik III und UniversitätsCentrum für Autoimmun- und Rheumatische Erkrankungen (UCARE) Universitätsklinikum und Medizinische Fakultät Carl Gustav Carus, Technische Universität Dresden, Dresden, Deutschland; 5 Rheumapraxis Templin, Templin, Deutschland; 6 Klinik für Innere Medizin III – Rheumatologie/Osteologie, Universitätsklinikum Jena, Jena, Deutschland; 7 Rheumatologie, MVZ MED BAYERN OST, Burghausen, Deutschland; 8 Medizinische Klinik und Poliklinik II, Universitätsklinikum Würzburg, Würzburg, Deutschland; 9 Zentrum für Interdisziplinäre Rheumatologie, Klinische Immunologie und Autoimmunerkrankungen (INDIRA), Department für Innere Medizin II, Universitätsklinik Tübingen, Tübingen, Deutschland; 10 Rheumatologisches Versorgungszentrum Steglitz, Berlin, Deutschland; 11 Ruhr-Universität Bochum, Bochum, Deutschland; 12 Rheumazentrum Ruhrgebiet, Herne, Deutschland; 13 Rheumazentrum Ratingen, Rheumatologische Gemeinschaftspraxis, Ratingen, Deutschland; 14 Rheumatologie, Klinische Immunologie und Osteologie, Immanuel-Krankenhaus Berlin, Berlin, Deutschland; 15 Klinik für Rheumatologie, Medizinische Fakultät, Universitätsklinikum Düsseldorf, Heinrich-Heine-Universität Düsseldorf, Düsseldorf, Deutschland; 16 Hiller Forschungszentrum Rheumatologie, Medizinische Fakultät, Universitätsklinikum Düsseldorf, Heinrich-Heine-Universität Düsseldorf, Düsseldorf, Deutschland; 17 Zentrum für Digitale Medizin, Medizinische Fakultät, Heinrich-Heine-Universität Düsseldorf, Düsseldorf, Deutschland; 18 ICH MVZ Grindel, Grindel, Deutschland; 19 Rheumapraxis München-Pasing, München, Deutschland; 20 Rheumateam Lahn-Dill-Siegerland – Wetzlar/Burbach, Wetzlar/Burbach, Deutschland; 21 Rheumatologische Schwerpunktpraxis Berlin, Berlin, Deutschland

**Keywords:** Versorgungsforschung, Rheumatische Erkrankungen, Langzeitbeobachtung, Medikamentöse Schmerztherapie, Alter, Health services research, Rheumatic diseases, Long-term observational research, Pharmacological pain management, Age

## Abstract

**Hintergrund:**

Die Versorgung älterer Menschen mit entzündlich-rheumatischen Erkrankungen (ERE) gewinnt immer mehr an Bedeutung. Im Jahresbericht 2026 aus der Kerndokumentation werden Charakteristika von Patienten mit ERE im höheren Lebensalter sowie Entwicklungen in der medikamentösen Therapie dargestellt.

**Methodik:**

Patienten aus der ambulanten rheumatologischen Routineversorgung und ihre behandelnden Rheumatologen dokumentieren einmal jährlich Krankheitsverlauf, Therapien und Krankheitsfolgen. Es wurden Daten von 2010 bis 2024 berücksichtigt und nach Altersgruppen ausgewertet.

**Ergebnisse:**

Im Jahr 2024 wurden 13.416 Patienten aus 14 rheumatologischen Einrichtungen eingeschlossen, 52 % waren ≥ 60 Jahre alt. Die häufigsten ERE ab 60 Jahren waren rheumatoide Arthritis (RA 57 %), Psoriasis-Arthritis (PsA 12 %), axiale Spondyloarthritis (axSpA 7 %), Polymyalgia rheumatica (PMR 6 %) und Riesenzellarteriitis (RZA 5 %). Die mittlere Zahl an Komorbiditäten stieg mit zunehmendem Alter von 1,3 (18–39 Jahre) bis 4,4 (≥ 80 Jahre). Insbesondere Frauen hatten mit zunehmendem Alter mehr Funktionseinschränkungen und Schmerzen. Ältere Menschen erhielten häufiger Glukokortikoide und Analgetika, aber seltener Biologika als jüngere. Bei RA erhielten Patienten ≥ 80 Jahre im Vergleich zu 18- bis 39-jährigen Patienten z. B. 25 % (≥ 80) vs. 47 % [18–39] Biologika und 37 % vs. 13 % Glukokortikoide. Dennoch folgen die Veränderungen dem Gesamttrend. So ist der Einsatz von biologischen krankheitsmodifizierenden Antirheumatika (bDMARDs) seit 2010 bei den ≥ 80-jährigen RA-Patienten von 7 auf 25 % gestiegen, während Glukokortikoide von 63 % auf 37 % zurückgingen.

**Schlussfolgerung:**

Trotz der insgesamt noch immer deutlich niedrigeren Rate an Biologika-Therapie im höheren Lebensalter zeigen die Langzeitdaten der Kerndokumentation, dass Biologika auch bei älteren Patienten im Vergleich zu vorherigen Kalenderjahren häufiger verordnet werden.

**Graphic abstract:**

**Figure:**
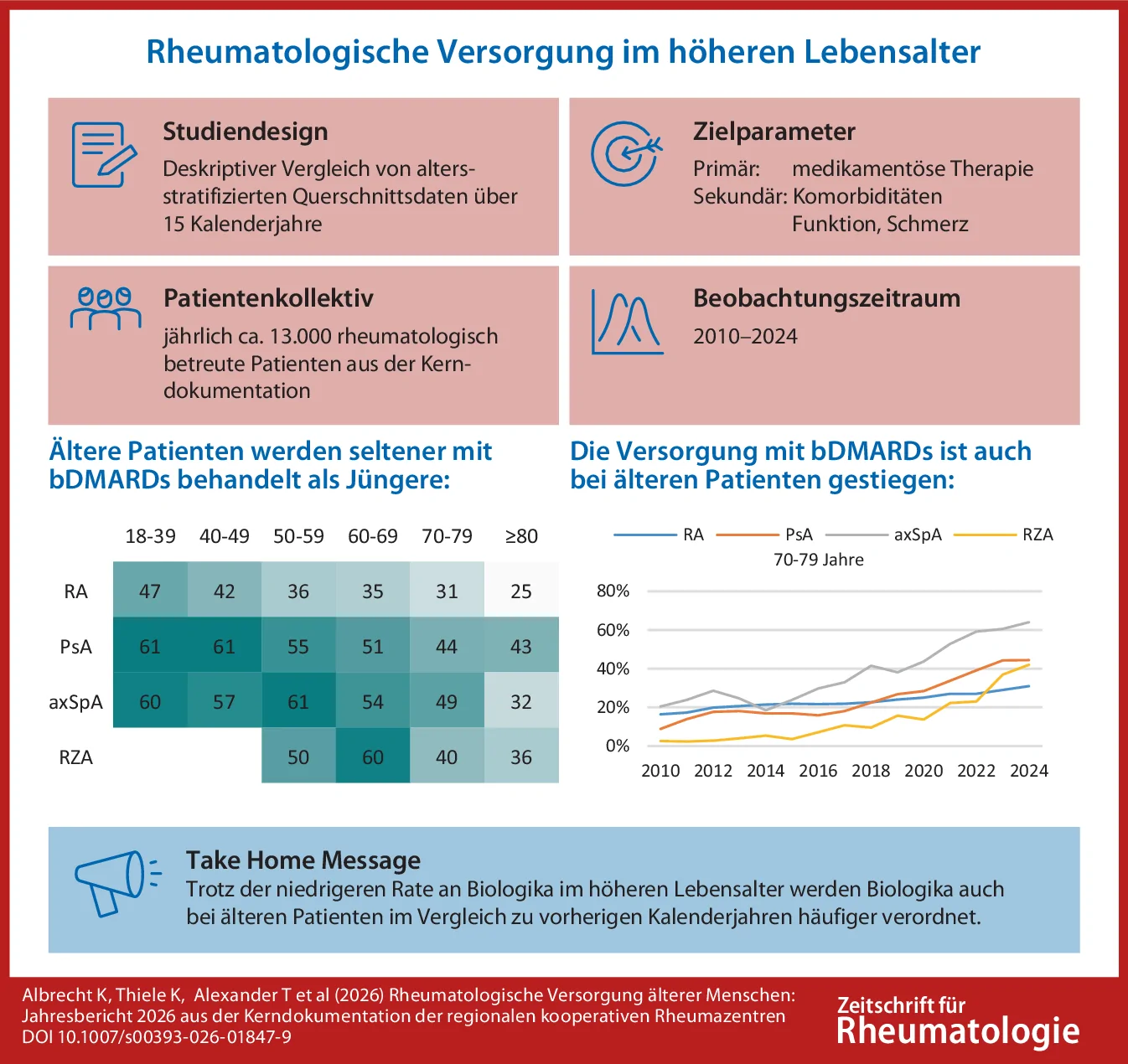

**Zusatzmaterial online:**

Die Online-Version dieses Beitrags (https://doi.org/10.1007/s00393-026-01847-9) enthält Tab. S1 und Abb. S1–S3.

## Abstract (en)

**Background:**

The rheumatological management of older people with inflammatory rheumatic diseases (iRMD) is becoming increasingly more important. The 2026 annual report from the National Database (NDB) of the Collaborative Arthritis Centers focusses on characteristics of older patients with iRMD as well as on developments in drug therapy.

**Methods:**

Within the NDB, patients under routine outpatient rheumatology care and the treating rheumatologists document disease outcomes, progression and treatment once a year. Data from 2010–2024 were included and analyzed by age groups.

**Results:**

In 2024 a total of 13,416 patients from 14 rheumatology centers were included; 52% were ≥ 60 years old. The most common iRMD in patients aged ≥ 60 years were rheumatoid arthritis (RA 57%), psoriatic arthritis (PsA 12%), axial spondyloarthritis (axSpA 7%), polymyalgia rheumatica (PMR 6%) and giant cell arteritis (GCA 5%). The mean number of comorbidities increased with age, from 1.3 (18–39 years) to 4.4 (≥ 80 years). Women experienced more functional limitations and more pain in higher age groups. Older patients more frequently received glucocorticoids and analgesics but received biologics less frequently than younger adults. For example, RA patients ≥ 80 years, as compared to patients aged 18–39 years, received 25% (≥80 years) vs. 47% (18–39 years) biologics and 37% vs. 13% glucocorticoids. Nevertheless, changes still followed the general trend. Since 2010, the use of biologic disease-modifying antirheumatic drugs (bDMARD) increased from 7% to 25% among RA patients aged ≥ 80 years, while glucocorticoids decreased from 63% to 37%.

**Conclusion:**

Despite overall lower use of biologics in older age groups, long-term data from the NDB show that biologics are being prescribed more frequently also to older patients compared to previous calendar years.

Die Versorgung älterer Menschen mit entzündlich-rheumatischen Erkrankungen (ERE) gewinnt durch die demografische Entwicklung immer mehr an Bedeutung. In Deutschland waren im Jahr 2024 fast 19 Mio. Menschen bzw. 23 % der Bevölkerung 65 Jahre und älter. Für das Jahr 2050 prognostiziert das Bundesinstitut für Bevölkerungsforschung einen weiteren Anstieg und eine starke Zunahme hochaltriger Menschen über 80 Jahre [1]. Da sich viele ERE in den mittleren Lebensjahren manifestieren und in der Regel chronisch verlaufen, ist mit einer weiter steigenden Anzahl an Patienten zu rechnen. Durch eine zielgerichtete medikamentöse Therapie kann die Entzündungsaktivität bei vielen Patienten inzwischen sehr gut kontrolliert werden [2], so dass die Sterblichkeitsrate bei rheumatoider Arthritis (RA) in den letzten Jahrzehnten deutlich gesunken ist [3]. Diese beiden Faktoren führen dazu, dass immer mehr ältere Menschen mit ERE rheumatologisch versorgt werden müssen. Die mit dem Altersprozess einhergehende reduzierte Immunabwehr, eine zunehmende Zahl an Begleiterkrankungen sowie physische und kognitive Einschränkungen stellen Rheumatologen in der therapeutischen Versorgung älterer Menschen allerdings vor zusätzliche Herausforderungen [4, 5]. Der Jahresbericht 2026 aus der Kerndokumentation hat daher die Versorgung der älteren Menschen mit ERE als Schwerpunktthema. Es werden Entwicklungen in der Versorgung mit medikamentösen Therapien im höheren Lebensalter über die letzten 14 Jahre dargestellt.

## Methodik

### Datenquelle

In der Kerndokumentation der Regionalen Kooperativen Rheumazentren dokumentieren Patienten aus der ambulanten rheumatologischen Routineversorgung und ihre behandelnden Rheumatologen seit 1993 einmal im Jahr Therapien, Krankheitsverlauf und Krankheitsfolgen. Für diesen Bericht wurden Daten aus den Jahren 2010–2024 berücksichtigt und nach Altersgruppen (18–39; 40–49; 50–59; 60–69; 70–79; ≥ 80 Jahre) und Diagnosen ausgewertet. Folgende ERE wurden berücksichtigt: rheumatoide Arthritis (RA), Psoriasis-Arthritis (PsA), axiale Spondyloarthritis (axSpA), systemischer Lupus erythematodes (SLE), systemische Sklerose (SSc), Sjögren-Erkrankung (SjD), idiopathische inflammatorische Myositiden (IIM), Mischkollagenosen („mixed connective tissue diseases“, MCTD), Polymyalgia rheumatica (PMR), Riesenzellarteriitis (RZA), ANCA (antineutrophile zytoplasmatische Antikörper) assoziierte Vaskulitiden (AAV), Morbus Behçet („Behçet disease“, BD), das adulte Still-Syndrom („adult onset Still’s disease“, AOSD) und autoinflammatorische Erkrankungen (AIE).

### Datenaufbereitung

Eingeschlossen wurden alle Dokumentationen mit einer entzündlich-rheumatischen Diagnose und vollständig dokumentierten Pflichtangaben (Geburtsjahr, Geschlecht, medikamentöse Therapie). Bei mehreren Besuchen pro Kalenderjahr wurde die jeweils vollständigste Visite verwendet. Die Daten der teilnehmenden Einrichtungen werden jährlich auf formale Plausibilität und Vollständigkeit geprüft. Unplausible Werte werden im Rahmen der Qualitätsprüfung korrigiert oder ausgeschlossen.

### Erhobene Parameter

Von allen Patienten werden Alter, Geschlecht, Erkrankungsalter und Krankheitsdauer erfasst. Rheumatologen dokumentieren Entzündungsmarker (Blutsenkungsgeschwindigkeit [BSG in mm/h]) und/oder C‑reaktives Protein (CRP in mg/dl), Krankheitsaktivität, Komorbiditäten und Therapien. Die ärztlich eingeschätzte Krankheitsaktivität wird bei allen Krankheitsentitäten mit einer numerischen Rating-Skala (NRS) von 0–10 erfasst, wobei 0 keiner und 10 der höchsten Aktivität entspricht. Medikamentöse Therapien umfassen Glukokortikoide (GC), konventionell-synthetische (cs), biologische (b) und zielgerichtete („targeted synthetic“, ts) immunmodulierende Basismedikamente („disease-modifying antirheumatic drugs“, DMARDs), nichtsteroidale Antirheumatika (NSAR), nichtopioidhaltige Analgetika und Opioide, Osteoporose-Prophylaxe und Osteoporose-Therapie.

Komorbiditäten werden mit ICD-10-GM (International Conference on Harmonisation, 10th edition German Modification) Codes erfasst und kategorisiert, u. a. Herz‑, Lungen‑, Leber‑, Magen-Darm‑, Nieren- und Schilddrüsenerkrankungen, Anämien, Diabetes mellitus, maligne Neoplasien, Osteoporose sowie degenerative Gelenk- und Wirbelsäulenerkrankungen. Neben der Anzahl an Komorbiditäten wird der Rheumatic Disease Comorbidity Index (RDCI, 0–9; [6]) abgebildet. Der RDCI wurde zur Quantifizierung der Komorbiditätslast bei rheumatischen Erkrankungen entwickelt und umfasst Herz-Kreislauf- und Lungenerkrankungen (jeweils 2‑fach gewichtet), Magen- und Krebserkrankungen, Diabetes mellitus, Frakturen und Depressionen.

Die Funktionsfähigkeit wird bei RA Patienten mit dem Funktionsfragebogen Hannover (FFbH) [7] und bei axSpA und PsA mit dem Bath Ankylosing Spondylitis Functional Index (BASFI; [8]) erhoben. Eine eingeschränkte Funktion ist definiert als FFbH < 70 bzw. BASFI > 2. Patienten geben die Schmerzintensität der letzten 7 Tage auf einer NRS von 0 (kein) bis 10 (stärkster) Schmerz an.

## Ergebnisse

### Charakteristika

Seit 2010 wurden jedes Jahr etwa 13.000 Patienten bundesweit dokumentiert. Im Jahr 2024 wurden 13.416 Patienten aus 14 Einrichtungen eingeschlossen. Die häufigsten Diagnosen ab 60 Jahre waren RA (57 %), PsA (12 %), axSpA (7 %), PMR (6 %) und RZA (5 %), gefolgt von SLE (4 %), AAV (3 %), SSc (2 %) und SjD (1 %). Von allen Patienten mit ERE waren 7023 (52 %) 60 Jahre und älter, 65 % waren Frauen, ab 70 Jahren 67 % und ab 80 Jahren 68 %. Die Krankheitsdauer lag ab 40 Jahren bei 11–12 Jahren im Median. Die mittlere Krankheitsaktivität betrug 1,5 ± 1,7 auf der NRS von 0–10 und lag in den Altersgruppen ab 60 Jahre bei 1,5 ± 1,7 bis 1,2 ± 1,5. Die mittlere BSG stieg mit zunehmendem Alter von 10,8 (18–39 Jahre) auf 15,7 (≥ 80 Jahre), während CRP-Werte in allen Altersgruppen im Median bei 0,5–0,6 mg/dl lagen.

Das mittlere Erkrankungsalter und die Altersverteilung unterscheiden sich bei den jeweiligen Krankheitsentitäten sehr. Bei RA, AAV, PMR, RZA und IIM waren über 50 % der Patienten 60 Jahre und älter (s. Anhang, Abb. S1). Betrachtet man die Diagnosen in den jeweiligen Altersgruppen, steigt der Anteil an RA-Patienten mit zunehmendem Alter an.

### Komorbiditäten

Die mittlere Zahl an Komorbiditäten reichte von 1,3 ± 1,3 (18–39 Jahre) bis 4,4 ± 2,3 (≥ 80 Jahre), der RDCI von 1,3 ± 1,4 (18–39 Jahre) bis 2,0 ± 1,5 (≥ 80 Jahre). Am häufigsten wurden arterielle Hypertonie, degenerative Gelenk- und Wirbelsäulenerkrankungen, koronare Herzkrankheit (KHK)/andere Herzerkrankungen und Osteoporose dokumentiert. Im Vergleich zu 2010 wurden 2024 häufiger Komorbiditäten dokumentiert: 2010 lag die mittlere Zahl an Komorbiditäten bei 2,4 ± 2,1 und reichte von 1,0 ± 1,1 (18–39 Jahre) bis 3,8 ± 2,3 (≥ 80 Jahre). Osteoporose (17 % in 2010, 13 % in 2024) und osteoporotische Frakturen (7 % in 2010, 4 % in 2024) sind etwas rückläufig, altersstratifizierte Werte sind im Anhang berichtet (Tabelle S1).

### Funktionseinschränkungen und Schmerzen

Von 5622 RA-Patienten hatten 38 % Funktionseinschränkungen gemäß FFbH < 70, in Abhängigkeit vom Alter 11 % (18–39 Jahre) bis 56 % (≥ 80 Jahre). Bei Frauen waren die Einschränkungen im Alter stärker als bei Männern (Abb. S2 im Anhang). Im Vergleich zu 2010 hat sich die Häufigkeit von Funktionseinschränkungen kaum verändert. Von 2456 Patienten mit SpA (axSpA und PsA) hatten 57 % einen BASFI > 2; 34 % der 18- bis 39-Jährigen und 77 % der ≥ 80-Jährigen (Tab. 1).

**Tab. 1:** Charakteristika aller Patienten mit entzündlich-rheumatischen Erkrankungen (ERE), Erhebungsjahr 2024

	Gesamt	18–39	40–49	50–59	60–69	70–79	≥ 80 J
*N*	13.416	1754	1719	2920	3554	2292	1177
– Davon RA, *N*	6331 (47 %)	448 (26 %)	548 (32 %)	1321 (45 %)	1910 (54 %)	1350 (59 %)	754 (64 %)
– PsA, *N*	1875 (14 %)	280 (16 %)	278 (16 %)	467 (16 %)	528 (15 %)	243 (11 %)	79 (7 %)
– axSpA, *N*	1814 (14 %)	466 (27 %)	396 (23 %)	461 (16 %)	340 (10 %)	114 (5 %)	37 (3 %)
– PMR, *N*	499 (4 %)	–	5 (0 %)	59 (2 %)	144 (4 %)	187 (8 %)	104 (9 %)
– RZA, *N*	390 (3 %)	–	1 (0 %)	26 (1 %)	88 (2 %)	157 (7 %)	118 (10 %)
Weiblich (%)	65 %	65 %	65 %	64 %	65 %	67 %	68 %
Krankheitsdauer, Jahre, Median [IQR]	11,0 [5,0–20,0]	8,5 [4,2–14,0]	11,0 [5,6–19,0]	11,8 [5,2–22,0]	11,0 [5,4–21,0]	12,0 [5,1–24,0]	11,4 [5,8–23,5]
Krankheitsaktivität, NRS 0–10, MW ± SD	1,5 ± 1,7	1,4 ± 1,7	1,5 ± 1,7	1,6 ± 1,7	1,5 ± 1,7	1,3 ± 1,5	1,2 ± 1,5
BSG, mm/h, Median [IQR] (*N*)	9,0 [5,0–18,0] (10.575)	7,0 [3,0–14,0] (1248)	8,0 [4,0–15,0] (1295)	8,0 [4,0–16,0] (2325)	9,0 [5,0–18,0] (2892)	11,0 [5,0–20,0] (1859)	11,0 [5,0–20,0] (956)
CRP, mg/dl, Median [IQR] (*N*)	0,3 [0,1–0,6] (11.376)	0,2 [0,1–0,5] (1335)	0,2 [0,1–0,6] (1393)	0,2 [0,1–0,5] (2505)	0,3 [0,1–0,6] (3087)	0,3 [0,1–0,6] (2018)	0,3 [0,1–0,6] (1038)
Mittlere Anzahl Komorbiditäten ± SD	2,9 ± 2,2	1,3 ± 1,3	1,9 ± 1,7	2,7 ± 1,9	3,3 ± 2,1	4,0 ± 2,2	4,4 ± 2,3
RDCI, MW ± SD	1,3 ± 1,4	0,4 ± 0,8	0,7 ± 1,1	1,1 ± 1,3	1,4 ± 1,4	1,8 ± 1,4	2,0 ± 1,5
FFbH, MW ± SD (nur RA)	73,2 ± 25,0	88,8 ± 16,6	83,0 ± 21,8	76,5 ± 23,2	72,4 ± 23,9	69,2 ± 25,7	60,4 ± 27,1
FFbH < 70, nur RA (*N*)	38 % (5622)	11 % (392)	22 % (480)	33 % (1170)	40 % (1702)	45 % (1211)	56 % (667)
BASFI > 2; axSpA, PsA (*N*)	57 % (2456)	34 % (569)	47 % (466)	67 % (635)	68 % (518)	76 % (203)	77 % (65)

*RA* rheumatoide Arthritis, *PsA* Psoriasis-Arthritis, *axSpA* axiale Spondyloarthritis, *PMR* Polymyalgia rheumatica, *RZA* Riesenzellarteriitis, *MW* Mittelwert, *SD* Standardabweichung, *BSG* Blutsenkungsgeschwindigkeit, *CRP* C-reaktives Protein, *IQR* Interquartilsabstand, *FFbH* Funktionsfragebogen Hannover, *BASFI* Bath Ankylosing Functional Index, *NRS* numerische Rating-Skala, *RDCI* Rheumatic Diseases Comorbidity Index

Ältere Frauen gaben häufiger moderate Schmerzen an als jüngere. Die Angaben von 2010 und 2024 sind vergleichbar. Männer gaben 2024 etwas seltener Schmerzen an als 2010 (Abb. S2 im Anhang).

### Medikamentöse Therapien

Patienten höheren Alters erhielten seltener bDMARDs als jüngere: Im Jahr 2024 wurden bei RA 25 % (≥ 80 Jahre) und 47 % (18–39 J.) mit bDMARDs behandelt. Ein deutlicher Unterschied zeigte sich vor allem bei TNFi mit 19 % (≥ 80 J.) vs. 37 % (18–39 J.), wie in Tab. 2 aufgeführt. Januskinase-Inhibitoren (JAKi) wurden mit 7 % (≥ 80 J.) vs. 14 % (40–49 J.) seltener verordnet. Gleichzeitig hatten ältere RA-Patienten häufiger Glukokortikoide (GC) mit 37 % (≥ 80 J.) vs. 13 % (18–39 J.), Analgetika (27 % vs. 7 %) und Opioide (10 % vs. 2 %) erhalten. Nichtsteroidale Antirheumatika (NSAR) wurden 40 % (18–39 J.) vs. 28 % (≥ 80 J.) verordnet, überwiegend als Bedarfsmedikation.

**Tab. 2:** Medikamentöse Therapie ausgewählter Diagnosen nach Altersgruppen

		Gesamt	18–39	40–49	50–59	60–69	70–79	≥ 80 Jahre
RA	csDMARDs	3957 (63 %)	221 (49 %)	328 (60 %)	846 (64 %)	1230 (64 %)	862 (64 %)	470 (62 %)
	bDMARDs	2204 (35 %)	211 (47 %)	231 (42 %)	471 (36 %)	678 (35 %)	424 (31 %)	189 (25 %)
	– TNFi	1429 (23 %)	164 (37 %)	159 (29 %)	305 (23 %)	421 (22 %)	258 (19 %)	122 (16 %)
	– IL-6i	307 (5 %)	28 (6 %)	35 (6 %)	57 (4 %)	105 (5 %)	58 (4 %)	24 (3 %)
	– Rituximab	207 (3 %)	5 (1 %)	15 (3 %)	46 (3 %)	69 (4 %)	52 (4 %)	20 (3 %)
	– Abatacept	206 (3 %)	11 (2 %)	16 (3 %)	48 (4 %)	69 (4 %)	43 (3 %)	19 (3 %)
	JAKi	675 (11 %)	38 (8 %)	77 (14 %)	172 (13 %)	213 (11 %)	119 (9 %)	56 (7 %)
	NSAR	2514 (40 %)	172 (38 %)	242 (44 %)	621 (47 %)	786 (41 %)	485 (36 %)	208 (28 %)
	– Bei Bedarf	1826 (74 %)	139 (82 %)	173 (72 %)	478 (79 %)	566 (74 %)	338 (72 %)	132 (65 %)
	GC	1551 (24 %)	59 (13 %)	119 (22 %)	263 (20 %)	461 (24 %)	373 (28 %)	276 (37 %)
	≥ 5 mg/d	871 (14 %)	42 (9 %)	78 (14 %)	162 (12 %)	269 (14 %)	198 (15 %)	122 (16 %)
	Analgetika	1144 (18 %)	30 (7 %)	65 (12 %)	214 (16 %)	350 (18 %)	279 (21 %)	206 (27 %)
	Opioide	441 (7 %)	11 (2 %)	26 (5 %)	73 (6 %)	142 (7 %)	110 (8 %)	79 (10 %)
PsA	csDMARDs	706 (38 %)	77 (28 %)	87 (31 %)	174 (37 %)	226 (43 %)	108 (44 %)	34 (43 %)
	bDMARDs	1010 (54 %)	171 (61 %)	170 (61 %)	257 (55 %)	270 (51 %)	108 (44 %)	34 (43 %)
	– TNFi	479 (26 %)	87 (31 %)	77 (28 %)	100 (21 %)	133 (25 %)	62 (26 %)	20 (25 %)
	– IL-17i	369 (20 %)	55 (20 %)	65 (23 %)	111 (24 %)	101 (19 %)	27 (11 %)	10 (13 %)
	– IL-12/23i	162 (9 %)	29 (10 %)	29 (10 %)	48 (10 %)	36 (7 %)	16 (7 %)	4 (5 %)
	JAKi	65 (3 %)	11 (4 %)	10 (4 %)	21 (4 %)	16 (3 %)	5 (2 %)	2 (3 %)
	GC	134 (7 %)	15 (5 %)	14 (5 %)	27 (6 %)	43 (8 %)	24 (10 %)	11 (14 %)
	≥ 5 mg/d	71 (4 %)	9 (3 %)	11 (4 %)	16 (3 %)	19 (4 %)	11 (5 %)	5 (6 %)
	NSAR	804 (43 %)	115 (41 %)	122 (44 %)	223 (48 %)	228 (43 %)	87 (36 %)	29 (37 %)
	– Bei Bedarf	561 (70 %)	93 (81 %)	87 (71 %)	155 (70 %)	152 (67 %)	57 (66 %)	17 (59 %)
	Analgetika	305 (16 %)	25 (9 %)	40 (14 %)	81 (17 %)	90 (17 %)	54 (22 %)	15 (19 %)
	Opioide	132 (7 %)	6 (2 %)	16 (6 %)	35 (7 %)	43 (8 %)	23 (9 %)	9 (11 %)
axSpA	NSAR	1155 (64 %)	315 (68 %)	269 (68 %)	309 (67 %)	196 (58 %)	50 (44 %)	16 (43 %)
	– Bei Bedarf	855 (76 %)	245 (79 %)	197 (75 %)	228 (77 %)	140 (74 %)	34 (74 %)	11 (79 %)
	bDMARDs	1110 (61 %)	292 (63 %)	238 (60 %)	295 (64 %)	197 (58 %)	73 (64 %)	15 (41 %)
	– TNFi	853 (47 %)	234 (50 %)	187 (47 %)	221 (48 %)	147 (43 %)	54 (47 %)	10 (27 %)
	– IL-17i	218 (12 %)	55 (12 %)	44 (11 %)	66 (14 %)	36 (11 %)	14 (12 %)	3 (8 %)
	MTX	165 (9 %)	18 (4 %)	22 (6 %)	53 (11 %)	44 (13 %)	19 (17 %)	9 (24 %)
	GC	84 (5 %)	9 (2 %)	8 (2 %)	29 (6 %)	26 (8 %)	5 (4 %)	7 (19 %)
	≥ 5 mg/d	50 (3 %)	8 (2 %)	7 (2 %)	18 (4 %)	11 (3 %)	3 (3 %)	3 (8 %)
	Analgetika	284 (16 %)	38 (8 %)	47 (12 %)	95 (21 %)	67 (20 %)	26 (23 %)	11 (30 %)
	Opioide	142 (8 %)	15 (3 %)	21 (5 %)	44 (10 %)	38 (11 %)	18 (16 %)	6 (16 %)
RZA	IL-6i	172 (44 %)	–	–	13 (50 %)	53 (60 %)	63 (40 %)	43 (36 %)
	MTX	76 (19 %)	–	–	6 (23 %)	13 (15 %)	29 (18 %)	28 (24 %)
	GC	153 (39 %)	–	–	10 (38 %)	30 (34 %)	61 (39 %)	52 (44 %)
	≥ 5 mg/d	82 (21 %)	–	–	8 (31 %)	18 (20 %)	35 (22 %)	21 (18 %)

*N* = 39 Patienten mit axSpA erhielten bei Vorliegen weiterer Diagnosen ein anderes bDMARD als TNFi oder IL-17

*RA* rheumatoide Arthritis, *PsA* Psoriasis-Arthritis, *axSpA* axiale Spondyloarthritis, *RZA* Riesenzellarteriitis, *GC* Glukokortikoide, *MTX* Methotrexat, *IL* Interleukin, *TNFi* Tumornekrosefaktor-Inhibitor, *NSAR* nichtsteroidale Antirheumatika, *csDMARDs* konventionell synthetische krankheitsmodifizierende Antirheumatika

Bei PsA wurden TNFi in den Altersgruppen vergleichbar häufig eingesetzt, IL-17i und IL-23i mit zunehmendem Alter seltener. Bei axSpA erhielten die 60- bis 79-Jährigen genauso häufig wie die jüngeren Patienten einen TNFi. Erst ab 80 Jahren wurden nur noch 27 % mit TNFi behandelt.

Bei RZA wurden 60- bis 69-Jährige am häufigsten mit IL-6Ri behandelt. Die ≥ 80-Jährigen hatten am häufigsten eine GC-Therapie, wobei eine Dosis ≥ 5 mg am häufigsten unter den 50- bis 59-Jährigen war.

### Entwicklungen in der medikamentösen Therapie

**Abb. 1:**
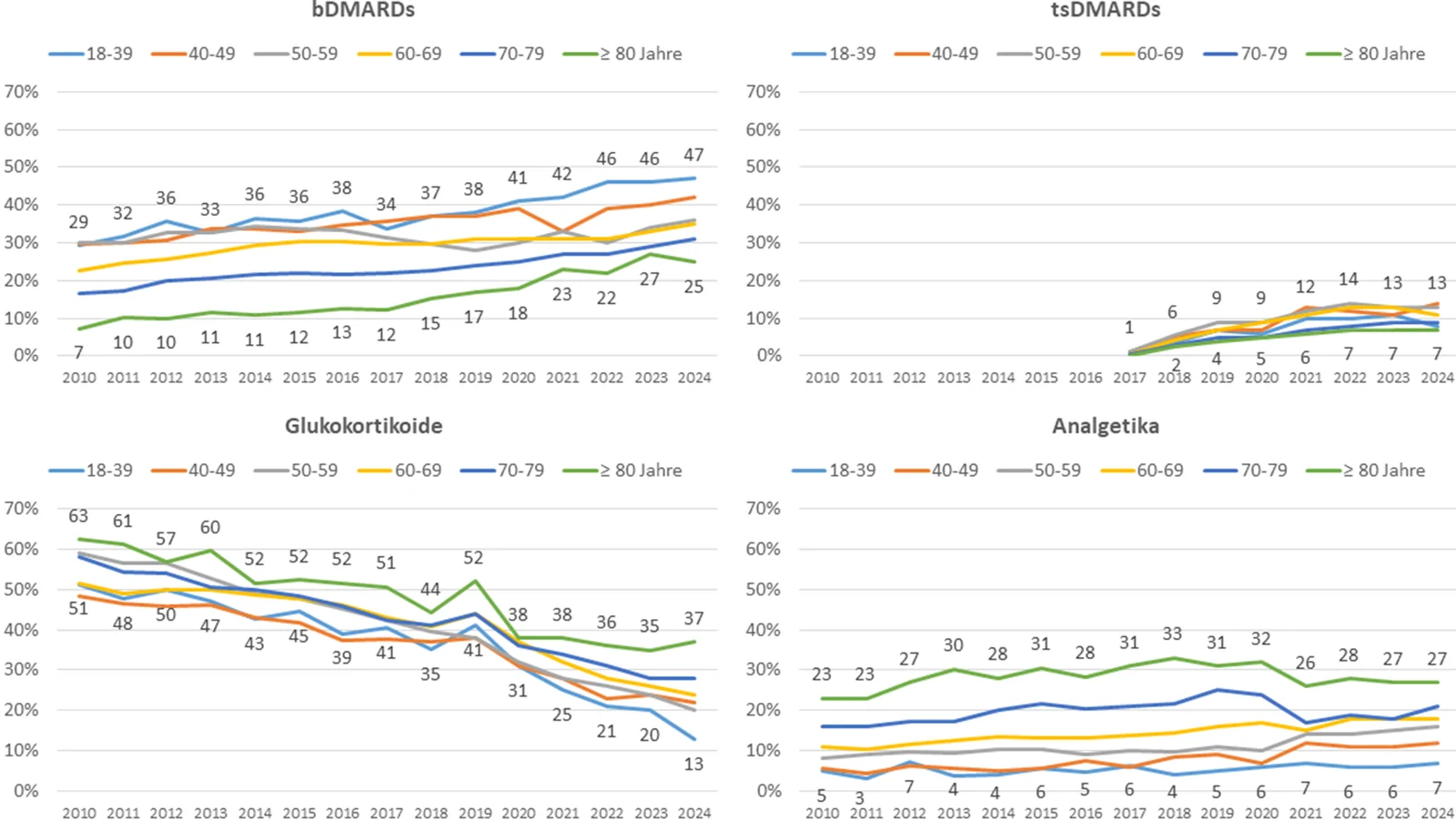
Therapie-Trends bei rheumatoider Arthritis 2010–2024. *bDMARDs* „biologic disease-modifying antirheumatic drugs“, *tsDMARDs* „targeted synthetic disease-modifying antirheumatic drugs“

Seit 2010 ist der Einsatz von bDMARDs bei RA in allen Altersgruppen gestiegen, bei den ≥ 80-Jährigen von 7 auf 25 % im Jahr 2024 (Abb. 1). Im gleichen Zeitraum gingen GC in dieser Altersgruppe von 63 auf 37 % zurück. Bei den tsDMARDs gab es in allen Altersgruppen einen leichten Anstieg. Wenig Veränderungen gab es bei nicht opioidhaltigen Analgetika und Opioiden. Eine Osteoporose-Therapie war in allen Altersgruppen der RA-Patienten rückläufig, bei den ≥ 80-Jährigen von 35 % in 2010 auf 25 % in 2024. Hingegen nahm die Osteoporose-Prophylaxe zu, von 55 auf 66 % in der gleichen Altersgruppe (Abb. S3 im Anhang).

**Abb. 2:**
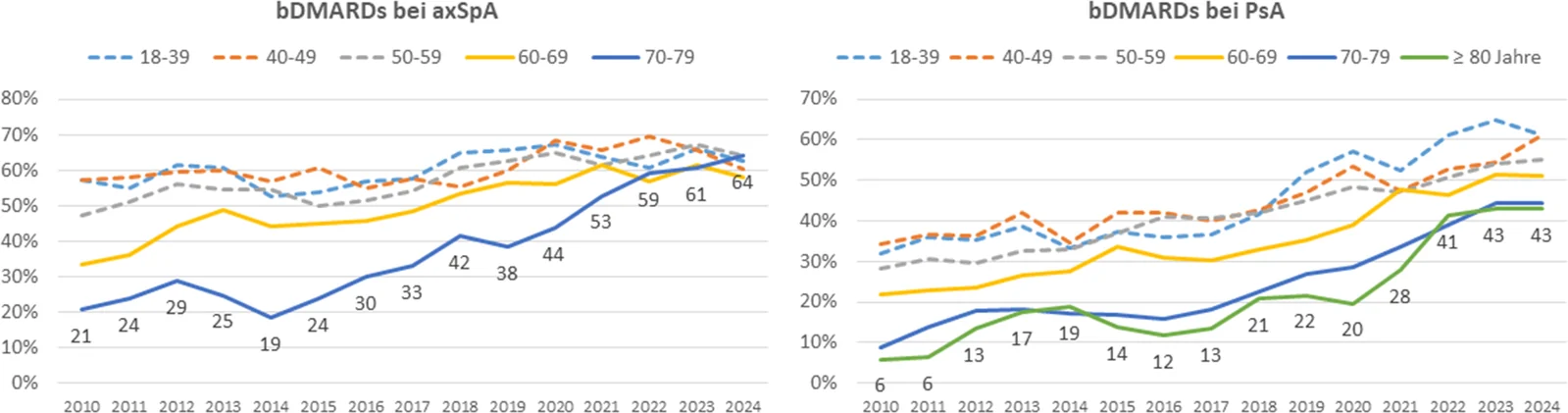
Therapie-Trends bei axialer Spondyloarthritis und Psoriasis-Arthritis 2010–2024. *axSpA* axiale Spondyloarthritis, *PsA* Psoriasis-Arthritis, *RZA* Riesenzellarteriitis, *GC* Glukokortikoide. (Die Altersgruppe ≥ 80 Jahre bei axSpA ist bei Fallzahlen < 20 nicht abgebildet)

Bei axSpA stiegen bDMARDs in der Altersgruppe 70–79 Jahre von 21 % in 2010 auf 64 % in 2024 und erreichte die Häufigkeit der jüngeren Altersgruppen (Abb. 2). In der Altersgruppe ≥ 80 Jahre waren die Fallzahlen zu klein für eine altersstratifizierte Auswertung. Auch bei PsA war der bDMARD Anstieg bei den 70- bis über 80-Jährigen groß.

**Abb. 3:**
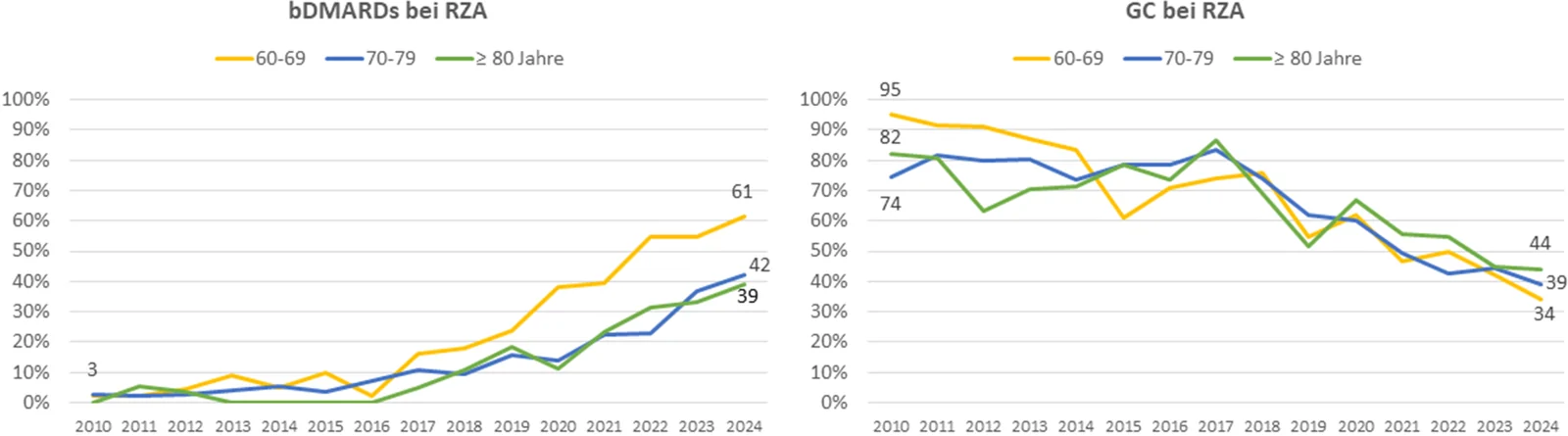
Therapie-Trends bei Riesenzellarteriitis 2010–2024. *RZA* Riesenzellarteriitis, *GC* Glukokortikoide. (Die Altersgruppe 50–59 Jahre ist bei Fallzahlen < 20 nicht abgebildet)

Bei RZA stieg die Häufigkeit von bDMARDs bis 2024 auf 61 % (60–69 Jahre), 42 % (70–79 Jahre) und 39 % (≥ 80 Jahre). Im gleichen Zeitraum ging die Verschreibung von GC stark zurück (Abb. 3).

## Diskussion

Etwas mehr als die Hälfte der Patienten, die in den rheumatologischen Einrichtungen der Kerndokumentation versorgt werden, sind heute 60 Jahre und älter. Die meisten älteren Patienten haben eine RA; weitere häufige Diagnosen in den höheren Altersgruppen sind PMR, RZA, PsA und axSpA. Insgesamt zeigt sich, dass ältere Patienten mit verschiedenen ERE seltener mit bDMARDs behandelt werden als jüngere. Allerdings zeigt der Vergleich mit Daten aus den letzten 15 Jahren eine eindrucksvolle Entwicklung zu deutlich häufigerer bDMARD-Behandlung auch in den höheren Altersgruppen. Bei RA hat sich der Anteil bei den Hochaltrigen mehr als verdreifacht, gleichzeitig benötigen immer weniger Patienten GC. Bei axSpA erhalten die 70- bis 79-Jährigen inzwischen vergleichbar häufig bDMARDs wie die Jüngeren, und auch bei PsA nähert sich die Häufigkeit der bDMARDs bei den Älteren den jüngeren Altersgruppen an. Eindrucksvoll ist auch der Anstieg der bDMARD-Therapie bei RZA und der deutliche Rückgang der GC. Diese Entwicklung repräsentiert das Potenzial einer spezialisierten fachärztlichen rheumatologischen Versorgung.

Bei den TNFi ist der altersabhängige Verordnungsunterschied bei PsA geringer als bei den Interleukin-Inhibitoren (ILi). Im Vergleich zu ILi werden TNFi bereits seit vielen Jahren angewendet. Studiendaten zu RA bestätigen, dass die Wirksamkeit und Therapiekontinuität von TNFi auch im hohen Lebensalter vergleichbar zu jüngeren Patienten ist [5]. Trotzdem werden TNFi gerade bei RA mit zunehmendem Alter weiterhin deutlich seltener eingesetzt. Eine JAKi Therapie haben etwa 11 % der RA Patienten. Patienten ab 70 Jahren erhielten bereits seit 2019 seltener JAKi, welches sich auch nach der Warnung der Europäischen Arzneimittel-Agentur (EMA) im Januar 2023 für Personen ab 65 Jahre [9] nicht geändert hat. Es ist anzunehmen, dass bereits vorher ein vorsichtiger Umgang mit JAKi bei älteren Patienten erfolgte.

Ein zurückhaltender Einsatz von b/tsDMARDs bei älteren Menschen kann dazu führen, dass die Krankheitskontrolle nicht ausreichend gelingt – der gleichzeitig erhöhte GC- und Analgetikabedarf, der sich diagnoseübergreifend zeigt, spricht dafür, auch wenn zusätzlich arthrotische Veränderungen den Schmerzmittelbedarf erhöhen dürften. In Kombination mit dem begrenzteren Einsatz gezielter Therapieansätze unterstützen die Ergebnisse daher die These, dass das höhere Alter nach wie vor eine Barriere für eine erfolgreiche *Treat-to-Target-Therapie* darstellt [10]. Der steigende Einsatz von bDMARDs auch bei den älteren Patienten deutet darauf hin, dass das Zutrauen in die Verträglichkeit und Sicherheit der Therapien gewachsen ist. Im RABBIT-Register ist die Kontinuität der b/tsDMARD-Therapie bei älteren und jüngeren Menschen vergleichbar [5]. Unabhängig von der Therapie haben ältere im Vergleich zu jüngeren Patienten ein höheres Risiko für die Entwicklung schwerwiegender Infektionen, Malignome und kardiovaskulärer Ereignisse [5], welche durch eine abnehmende Immunkompetenz, mehr Komorbiditäten und weitere Alterungsprozessen begünstigt werden. Hingegen erhöht eine länger dauernde Therapie mit GC auch schon in relativ niedrigen Dosierungen nachweisbar das Risiko für schwerwiegende Infektionen, insbesondere in Kombination mit den im Alter häufigen Komorbiditäten wie z. B. Diabetes, chronisch-obstruktive Lungenerkrankung (COPD) oder Nierenerkrankung sowie bei schlecht kontrollierter Krankheitsaktivität [5]. Das Einsparpotenzial von GC durch bDMARDs zeigt sich gerade bei den RZA-Verlaufsdaten eindrucksvoll. Mit der Zulassung des ersten JAKi für die Therapie der RZA gibt es seit 2025 eine weitere Behandlungsoption, deren Entwicklung in den kommenden Jahren beobachtet werden kann.

Höheres Alter stellt nach wie vor eine Barriere für eine erfolgreiche Treat-to-Target-Therapi dar.

Erfreulich ist ein leichter Rückgang der Häufigkeit einer Osteoporose und einer Osteoporose-Therapie bei RA, bei zunehmender Prophylaxe. Hier kann durch das Einsparen von GC eine deutliche Verbesserung der Versorgung erreicht werden.

Die Häufigkeit der Multimorbidität im höheren Alter mit degenerativen Gelenk- und Wirbelsäulenerkrankungen, osteoporotische bzw. Sturzfrakturen, Krebserkrankungen oder auch Neuralgien, z. B. nach einer Herpes-Zoster-Infektion, sind zusätzliche Faktoren für erhöhten Schmerzmittelbedarf. Ältere Frauen geben häufiger moderate bis starke Schmerzen an als jüngere Patientinnen. NSAR werden aufgrund kardialer, gastrointestinaler und renaler Risiken im höheren Lebensalter vorsichtiger eingesetzt. Daher überrascht der relativ häufige Einsatz von NSAR bei RA über alle Altersgruppen, der allerdings oft bedarfsorientiert erfolgt. Mit steigendem Alter werden zunehmend zusätzlich Analgetika verordnet. Das häufigste in Deutschland verwendete Analgetikum ist Metamizol, auch bei Patienten mit ERE [11]. Dies birgt insbesondere ab dem 80. Lebensjahr und in Kombination mit Methotrexat ein erhöhtes Risiko für Agranulozytose [12]. Daher wird von dieser Kombination in den DGRh-Empfehlungen ab 80 Jahren abgeraten [13]. Opioide kommen zur vorübergehenden Behandlung starker Schmerzen oder bei unzureichendem Ansprechen auf andere Therapien in Betracht, gehen aber gerade für die älteren Patienten auch mit unerwünschten und relativ häufigen Risiken wie Obstipation, Übelkeit, Schläfrigkeit, Schwindel und Stürzen einher. Die Häufigkeit von Analgetika und Opioiden in den Altersgruppen ab 60 Jahre ist ebenso wie die Schmerzangaben seit 2010 konstant. Die neue S3-Leitlinie zum Schmerzmanagement bei geriatrischen Patienten in allen Versorgungssettings gibt evidenzbasierte Empfehlungen, die auf einen multimodalen Ansatz fokussieren [14]. Allerdings ist die Umsetzung im ambulanten Versorgungsalltag eine Herausforderung, ebenso wie eine nebenwirkungsarme medikamentöse Schmerztherapie. Für einige Patienten, die mit einem stationären Aufenthalt zurechtkommen, kann eine multimodale rheumatologische Komplexbehandlung [15] eine Option sein.

Die neue S3-Leitlinie zum Schmerzmanagement empfiehlt einen multimodalen Ansatz

Leider gibt die FFbH-Verteilung kein Anzeichen dafür, dass ältere Patienten heute weniger Funktionseinschränkungen haben als noch vor 14 Jahren. Der FFbH ist mit dem gängigen Schwellenwert von 70 aber auch kein gut geeignetes Instrument, um Veränderungen in der Funktionskapazität gerade bei Älteren abzubilden. Die Fragen sind so konzipiert, dass man sie auch ohne eine ERE ab einem gewissen Alter nur noch mit Einschränkungen beantworten kann (z. B. einen 10 l Wassereimer 10 m weit tragen, 30 min ohne Unterbrechung stehen) [7]. Berücksichtigt man altersgleiche Gruppen, kann der FFbH aber über die Kalenderjahre als Funktionsparameter herangezogen werden.

### Stärken und Limitationen

Die Kerndokumentation umfasst mit etwa 13.000 Patienten weniger als 1 % der geschätzten Zahl an Menschen mit ERE in Deutschland [16]. Alle Patienten befinden sich in fachärztlicher Versorgung ausgewiesener Rheumazentren mit hoher Expertise. Bei Patienten, die nicht rheumatologisch betreut werden ist die Häufigkeit von DMARD-Verordnungen deutlich niedriger [17, 18].

Neben der RA als häufigster Diagnose im Alter werden diesmal erstmals altersstratifizierte Daten aus der Kerndokumentation zu weiteren ERE wie axSpA, PsA und RZA berichtet. Die große Zahl der Patienten, die jedes Jahr in der Kerndokumentation erfasst werden, ermöglichen eine detaillierte Darstellung der Charakteristika und der medikamentösen Versorgung nach Altersgruppen. Die Therapien werden von allen Patienten verpflichtend erfasst. Dies gilt auch für Komorbiditäten, die ebenfalls ein Pflichtfeld sind. Im Vergleich zu Abrechnungsdaten [19] scheinen Komorbiditäten eher untererfasst, z. B. Hypertonie, Osteoporose oder Depressionen. Alle Einrichtungen dokumentieren Komorbiditäten, es gibt aber Unterschiede zwischen den Einrichtungen, von wem (Rheumatologen, Rheumatologische Fachassistenz, Dokumentare), wie (ICD-10-Codes, Liste, Freitext) und in welchem Umfang Komorbiditäten abgefragt und dokumentiert werden. Analgetika wurden bislang nicht spezifisch erfasst, aber es ist geplant, Metamizol zukünftig auch gesondert abzufragen. Patienten, die an der Kerndokumentation teilnehmen, befinden sich alle in rheumatologischer Versorgung. Erschwerte Zugangswege im Alter, z. B. durch Heimversorgung können nicht abgebildet werden, es gibt auch keine Daten zum Pflegegrad. Aus Kassendaten ist bekannt, dass nur noch wenige RA-Patienten nach Aufnahme in ein Pflegeheim rheumatologisch versorgt werden [20].

## Fazit für die Praxis

- Menschen mit entzündlich-rheumatischen Erkrankungen (ERE) benötigen auch im hohen Lebensalter eine ausreichend wirksame Therapie mit krankheitsmodifizierenden Antirheumatika (DMARD).
- Trotz der insgesamt deutlich niedrigeren Versorgung mit biologischen DMARDs (bDMARDs) im Vergleich zu jüngeren Patienten zeigen die 15-Jahres-Trend-Daten, dass inzwischen auch ältere Patienten häufiger mit bDMARDs behandelt werden und die Verwendung von Glukokortikoiden (GC) rückläufig sind.
- Der höhere Bedarf an GC und Analgetika bei vielen älteren Patienten und auch die Schmerzangaben der Betroffenen zeigen, dass die Versorgung von ERE im hohen Lebensalter verbessert werden könnte.
- Da Nebenwirkungsprofile die medikamentöse Schmerztherapie bei älteren Patienten erschweren, nehmen nichtmedikamentöse Behandlungsansätze einen wichtigen Stellenwert ein.

## Supplementary Information

**Tab. S1** Komorbiditäten nach Altersgruppen 2010 und 2024; **Abb. S1** Altersverteilung entzündlich-rheumatischer Erkrankungen; **Abb. S2** Funktionskapazität (FFbH) und Schmerzangaben (NRS 0–10) nach Altersgruppen und Geschlecht bei Rheumatoider Arthritis, 2010 und 2024; **Abb. S3** Osteoporose-Prophylaxe und Osteoporose-Therapie

## Data Availability

Die dem Artikel zugrunde liegenden Daten können durch Anfrage an die korrespondierende Autorin zur Verfügung gestellt werden.
