# Supplementary material for: Rheumatological care of older people: Annual report 2026 from the National Database of the regional Collaborative Arthritis Centers
Source: Z Rheumatol. 2026 Jul 16;85(7):578–87. [Article in German] doi: 10.1007/s00393-026-01847-9 (PMC13558291; doi:10.1007/s00393-026-01847-9)

Tab. S1 Komorbiditäten nach Altersgruppen 2010 und 2024

**2024**	**Gesamt**	**18-39**	**40-49**	**50-59**	**60-69**	**70-79**	**≥80 Jahre**
N	13,307	1,729	1,688	2,894	3,540	2,282	1,174
Mittlere Anzahl Komorbiditäten ± SD	2.9 ± 2.2	1.3 ± 1.3	1.9 ± 1.7	2.7 ± 1.9	3.3 ± 2.1	4.0 ± 2.2	4.4 ± 2.3
RDCI, MW ± SD	1.3 ± 1.4	0.4 ± 0.8	0.7 ± 1.1	1.1 ± 1.3	1.4 ± 1.4	1.8 ± 1.4	2.0 ± 1.5
Arterielle Hypertonie	4,481 (34%)	99 (6%)	251 (15%)	847 (29%)	1,448 (41%)	1,164 (51%)	672 (57%)
Degenerative Gelenkerkrankungen	3,640 (27%)	55 (3%)	163 (10%)	654 (23%)	1,262 (36%)	955 (42%)	551 (47%)
Degenerative Wirbelsäulenerkrankungen	3,463 (26%)	182 (11%)	288 (17%)	789 (27%)	1,032 (29%)	758 (33%)	414 (35%)
KHK/andere Herzerkrankungen	1,805 (14%)	53 (3%)	90 (5%)	249 (9%)	505 (14%)	549 (24%)	359 (31%)
Depression/Psychische Erkrankung	1,401 (11%)	120 (7%)	188 (11%)	373 (13%)	470 (13%)	169 (7%)	81 (7%)
Diabetes mellitus	1,165 (9%)	23 (1%)	57 (3%)	206 (7%)	379 (11%)	328 (14%)	172 (15%)
Lebererkrankungen	776 (6%)	52 (3%)	84 (5%)	163 (6%)	260 (7%)	157 (7%)	60 (5%)
Lungenerkrankungen	1,619 (12%)	110 (6%)	150 (9%)	334 (12%)	492 (14%)	371 (16%)	162 (14%)
Magen-/Darmerkrankungen	1,353 (10%)	88 (5%)	134 (8%)	290 (10%)	366 (10%)	317 (14%)	158 (13%)
Maligne Neoplasie	1,089 (8%)	27 (2%)	52 (3%)	178 (6%)	324 (9%)	312 (14%)	196 (17%)
Nierenerkrankungen	1,052 (8%)	44 (3%)	71 (4%)	131 (5%)	265 (7%)	299 (13%)	242 (21%)
Osteoporose	1,692 (13%)	20 (1%)	41 (2%)	174 (6%)	490 (14%)	550 (24%)	417 (36%)
Osteoporose mit Fraktur	539 (4%)	7 (0%)	7 (0%)	61 (2%)	127 (4%)	176 (8%)	161 (14%)
Schilddrüsenerkrankungen	2,120 (16%)	213 (12%)	220 (13%)	464 (16%)	610 (17%)	435 (19%)	178 (15%)
**2010**	**Gesamt**	**18-39**	**40-49**	**50-59**	**60-69**	**70-79**	**≥80 Jahre**
N	10,066	1,199	1,575	2,102	2,326	2,325	539
Mittlere Anzahl Komorbiditäten ± SD	2.4 ± 2.1	1.0 ± 1.1	1.5 ± 1.6	2.2 ± 1.8	2.8 ± 2.0	3.3 ± 2.1	3.8 ± 2.3
RDCI, MW ± SD	1.1 ± 1.3	0.3 ± 0.7	0.6 ± 1.0	0.9 ± 1.2	1.3 ± 1.3	1.6 ± 1.4	1.8 ± 1.4
Arterielle Hypertonie	3,415 (34%)	86 (7%)	279 (18%)	604 (29%)	992 (43%)	1,141 (49%)	313 (58%)
Degenerative Gelenkerkrankungen	2,281 (23%)	41 (3%)	163 (10%)	417 (20%)	681 (29%)	773 (33%)	206 (38%)
Degenerative Wirbelsäulenerkrankungen	1,683 (17%)	56 (5%)	148 (9%)	343 (16%)	480 (21%)	504 (22%)	152 (28%)
KHK/andere Herzerkrankungen	1,149 (11%)	31 (3%)	63 (4%)	138 (7%)	285 (12%)	481 (21%)	151 (28%)
Depression/Psychische Erkrankung	508 (5%)	56 (5%)	97 (6%)	147 (7%)	103 (4%)	91 (4%)	14 (3%)
Diabetes mellitus	1,012 (10%)	17 (1%)	56 (4%)	172 (8%)	288 (12%)	387 (17%)	92 (17%)
Lebererkrankungen	339 (3%)	22 (2%)	49 (3%)	85 (4%)	94 (4%)	76 (3%)	13 (2%)
Lungenerkrankungen	845 (8%)	39 (3%)	89 (6%)	189 (9%)	253 (11%)	225 (10%)	50 (9%)
Magen-/Darmerkrankungen	1,144 (11%)	49 (4%)	118 (7%)	248 (12%)	287 (12%)	341 (15%)	101 (19%)
Maligne Neoplasie	556 (6%)	9 (1%)	41 (3%)	82 (4%)	160 (7%)	223 (10%)	41 (8%)
Nierenerkrankungen	722 (7%)	49 (4%)	55 (3%)	85 (4%)	160 (7%)	284 (12%)	89 (17%)
Osteoporose	1,685 (17%)	30 (3%)	100 (6%)	245 (12%)	438 (19%)	663 (29%)	209 (39%)
Osteoporose mit Fraktur	690 (7%)	8 (1%)	31 (2%)	79 (4%)	164 (7%)	299 (13%)	109 (20%)
Schilddrüsenerkrankungen	914 (9%)	80 (7%)	126 (8%)	186 (9%)	258 (11%)	208 (9%)	56 (10%)

Abb. S1 Altersverteilung entzündlich-rheumatischer Erkrankungen

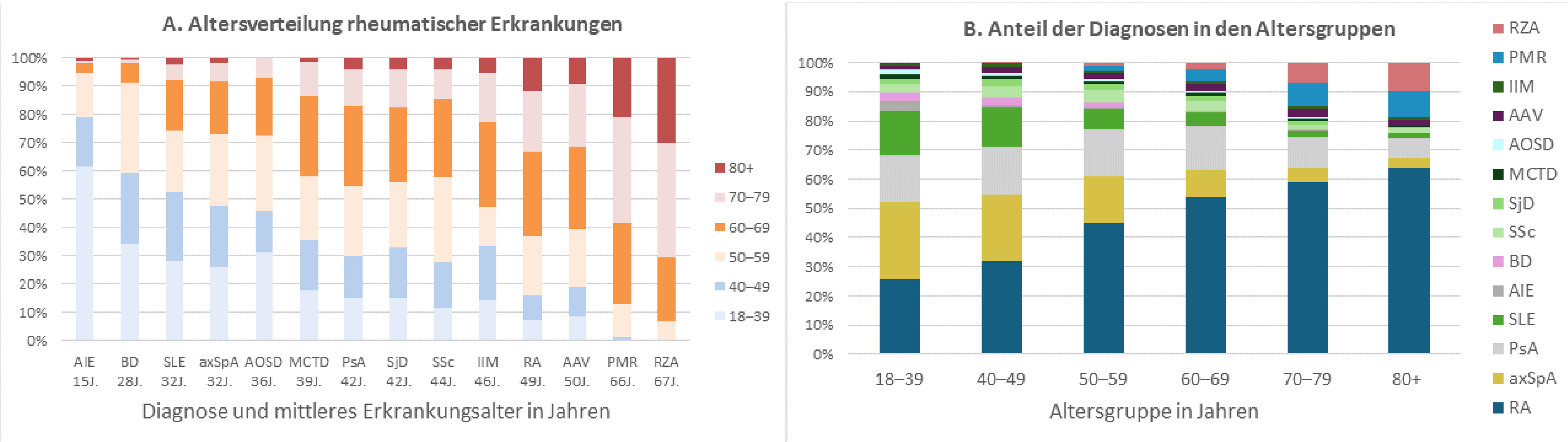

AIE Autoinflammatorische Erkrankungen, BD Behçet Erkrankung, SLE Systemischer Lupus erythematodes, axSpA axiale Spondyloarthritis, AOSD Adult Onset Still's Disease, MCTD Mixed Connective Tissue Disease, PsA Psoriasis-Arthritis, SjD Sjögren Erkrankung, SSc systemische Sklerose, IIM idiopathische inflammatorische Myositiden, RA Rheumatoide Arthritis, AAV ANCA-assoziierte Vaskulitiden, PMR Polymyalgia rheumatica, RZA Riesenzellarteriitis

Abb. S2 Funktionskapazität (FFbH) und Schmerzangaben (NRS 0-10) nach Altersgruppen und Geschlecht bei Rheumatoider Arthritis, 2010 und 2024

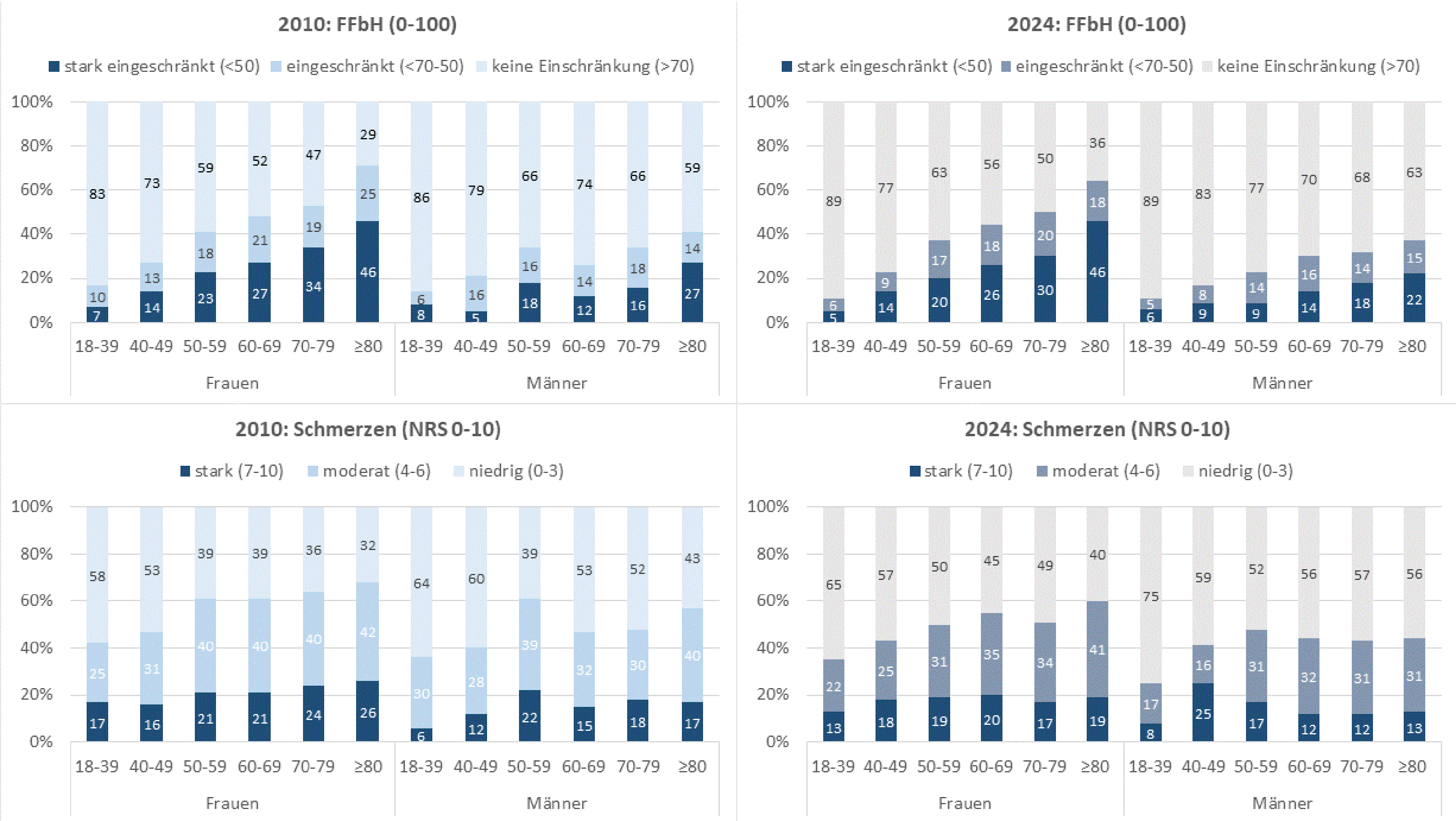

FFbH Funktionsfragebogen Hannover, NRS Numerische Rating Skala von 0 bis 10

Abb. S3 Osteoporose-Prophylaxe und Osteoporose-Therapie

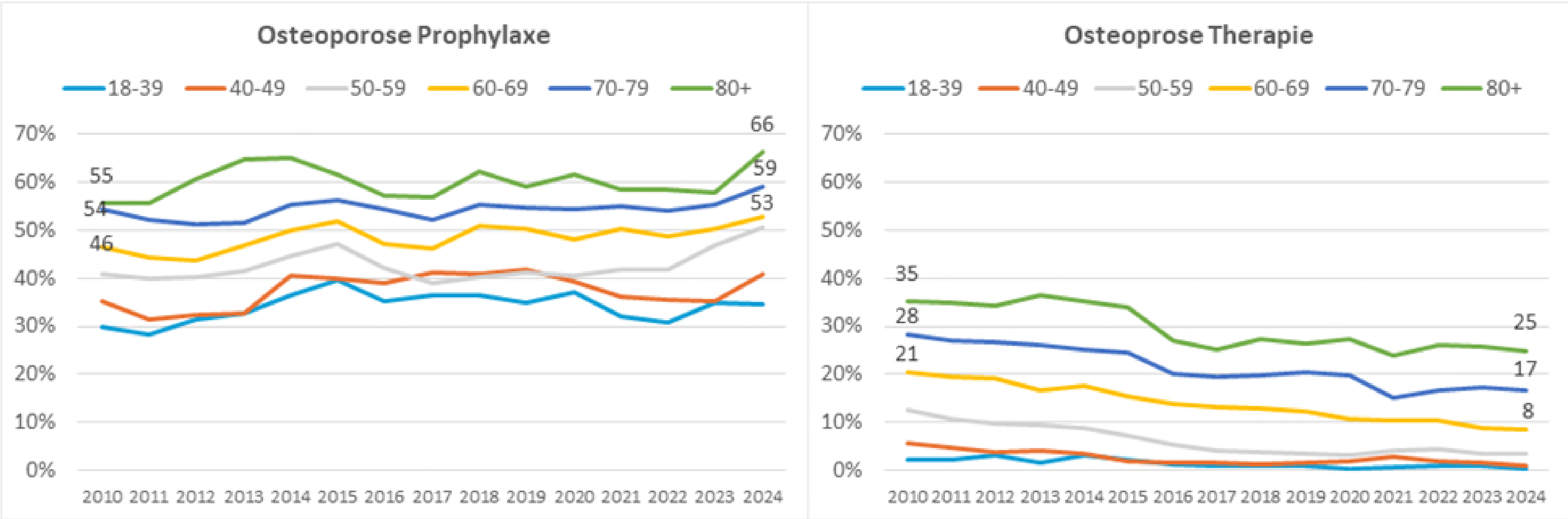

Supplement: Supplementary file 1 [file 393_2026_1847_MOESM1_ESM.pdf]
